# The role of IL-11 in chronic diseases

**DOI:** 10.3389/fimmu.2026.1763360

**Published:** 2026-02-18

**Authors:** Jingxuan Zhang, Yan Leng, Wenyuan Li, Bihan Wang, Baichuan Yang, Wei Li

**Affiliations:** Department of Anesthesiology, Renmin Hospital of Wuhan University, Wuhan, China

**Keywords:** chronic disease, cytokine signaling, fibrosis, il-11, inflammation, therapeutic targeting

## Abstract

**Background:**

Initially recognized for its role in hematopoiesis, Interleukin-11 (IL-11) is now understood to possess a wide spectrum of biological activities. It is involved in critical physiological processes, including immune cell differentiation, inflammatory response regulation, and tissue repair. Consequently, IL-11 is strongly implicated in the pathogenesis and progression of various chronic diseases.

**Discussion:**

This review examines the multifaceted biology of IL-11, detailing its discovery, molecular structure, and signaling mechanisms. We also explore its expression patterns across different tissues. A primary focus is placed on elucidating the critical role of IL-11 in the pathogenesis and progression of various chronic diseases. Furthermore, we discuss the emerging therapeutic potential of targeting the IL-11 pathway, evaluating evidence from both experimental models and clinical studies.

**Conclusion:**

By synthesizing current knowledge on the biological characteristics and disease associations of IL-11, this review aims to provide a comprehensive theoretical foundation for future research into its role in chronic diseases and its potential as a therapeutic target.

## 1 Introduction

Chronic diseases constitute a significant and growing global health burden and are the leading cause of decline in health status, aging, disability, and death (1). Interleukin-11 (IL-11), a crucial cytokine of the IL-6 family, includes not only IL-11 but also IL-6, IL-27, IL-31, leukemia inhibitory factor (LIF), ciliary neurotrophic factor (CNTF), cardiotrophin-like cytokine (CLC), oncostatin M (OSM), cardiotrophin-1 (CT-1), and neuropoietin (NPN/CT-2) (2). Initially, IL-11 was identified for its role in hematopoietic regulation, particularly in stimulating megakaryocytopoiesis and thrombopoiesis (3). However, subsequent research indicated that IL-11 possessed a broader range of biological activities, extensively participating in immune cell differentiation, regulation of inflammatory responses, and tissue repair, among many physiological processes (4, 5). These diverse functions position IL-11 as a potential mediator in the pathogenesis of various chronic diseases.

**Figure 1:**
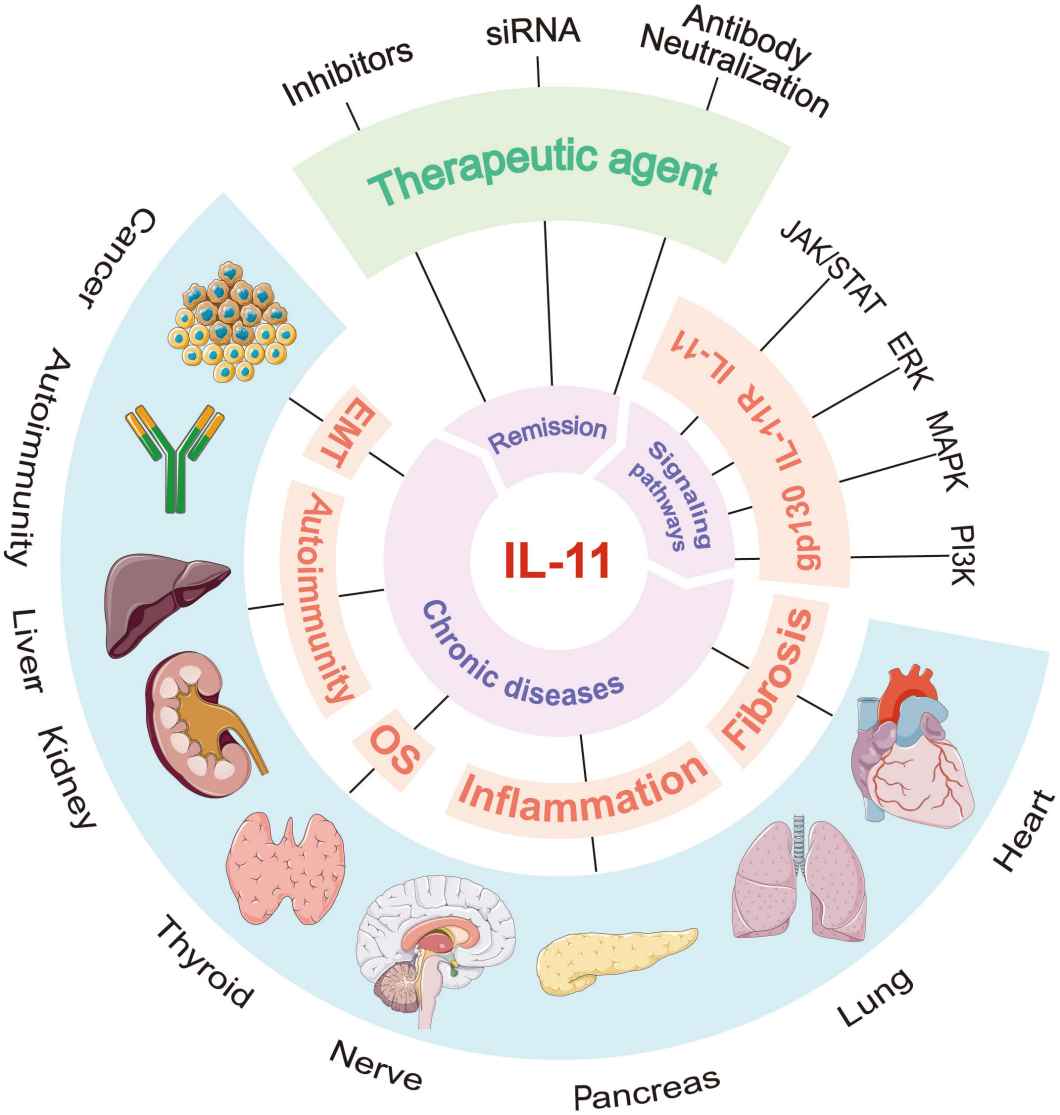
Overview of “IL-11 in chronic diseases”. IL-11 activates signaling pathways such as JAK/STAT, ERK, and PI3K/Akt by binding to its specific receptor IL-11Rα and the signal-transducing receptor gp130, thereby regulating cell proliferation, differentiation, and survival. In chronic diseases, IL-11 levels are significantly elevated and closely associated with the onset and progression of conditions like cardiovascular diseases, respiratory diseases, neurodegenerative diseases, endocrine disorders, kidney diseases, liver diseases, autoimmune diseases, and cancer. It plays a key role in fibrosis, inflammatory responses, and tissue damage. IL-11 inhibitors, siRNA targeting IL-11, and antibody-based neutralization of IL-11 have all played significant roles in treating related diseases.

This review will provide an in-depth analysis of the current understanding of IL-11’s role in chronic diseases. We will focus on the pathological and physiological functions of IL-11, as well as its molecular mechanisms and clinical significance in chronic diseases such as cardiovascular, respiratory, neurological, endocrine, kidney, liver, autoimmune diseases, and cancer, exploring potential therapeutic opportunities targeting the IL-11 signaling pathway for chronic diseases. By elucidating the complex biological functions of IL-11, this review aims to establish a robust theoretical framework for understanding its critical role in human health and longevity (**Figure 1**).

## 2 Biological properties of IL-11

### 2.1 Discovery and structure of IL-11

IL-11 was initially discovered in 1990 by Paul et al. in the supernatant of a primate marrow stromal cell line (Pu-34) culture (6). It was a novel hematopoietic cytokine capable of stimulating the proliferative activity of IL-6-dependent mouse plasmacytoma cell line T1165, even in the presence of anti-IL-6 monoclonal antibodies that neutralize IL-6. Subsequently, the gene for IL-11 was successfully cloned and expressed within same year (6).

Further research has revealed that IL-11 is a non-glycosylated secretory protein with a molecular weight of approximately 19 kDa and is composed of 178 amino acids. Its gene is located on human chromosomal locus 19q13, consisting of 5 exons and 4 introns (2). IL-11 possesses a typical type-1 four α-helical bundle structure (7). Advanced studies have utilized cryo-electron microscopy (cryo-EM) and crystallography methods to demonstrate the structure of the human IL-11 signaling complex, which includes the cytokine, homologous receptor (IL-11Rα), and the complete extracellular domain complex containing the shared IL-6 family β receptor gp130 (8). Through mutational analysis and molecular dynamics simulation, researchers have proposed a model of the IL-11-IL-11Rα complex, suggesting a 1:1:1 stoichiometry between IL-11, IL-11Rα, and gp130, with nanomolar-level binding affinity (9). The formation of the IL-11 signaling complex is a multi-step process involving conformational reorganization of IL-11 (8). The juxtamembrane domain of gp130 is dynamic, which is crucial for the activation of Janus kinase (JAK) molecules and the initiation of downstream signaling pathways (8). Additionally, the cytokine mutant IL-11 Mutein has been found to competitively inhibit signaling in human cell lines, blocking the formation of hexamers (8, 10). Notably, even though IL-11 and IL-6 both follow the canonical assembly logic of the gp130 cytokine family—where the cytokine first binds its cognate α receptor and subsequently recruits gp130 to form a higher-order signaling complex—IL-11 exhibits receptor-binding site features that differ from those of IL-6. Moreover, cryo-EM structures of the IL-11 signaling complex have revealed IL-11–specific gp130 domain dynamics as well as inhibitory stabilization mechanisms mediated by certain IL-11 cytokine variants, which are not fully congruent with the classical IL-6 assembly paradigm (8, 11).

### 2.2 IL-11 signaling pathways

IL-11 activates downstream signaling by binding to the IL-11-specific receptor IL-11Rα and the signaling receptor glycoprotein gp130 (11, 12). Initially, IL-11 interacts its specific receptor IL-11Rα at site I to form a 1:1 complex (8, 9). Subsequently, the IL-11–IL-11Rα complex engages with gp130 molecules at site II. Two trimer intermediates further interact through the D1 domain of gp130, binding at site III to form the final hexameric signaling complex. The hexamer is composed of two IL-11-IL-11Rα-gp130 molecules (8).

**Figure 2:**
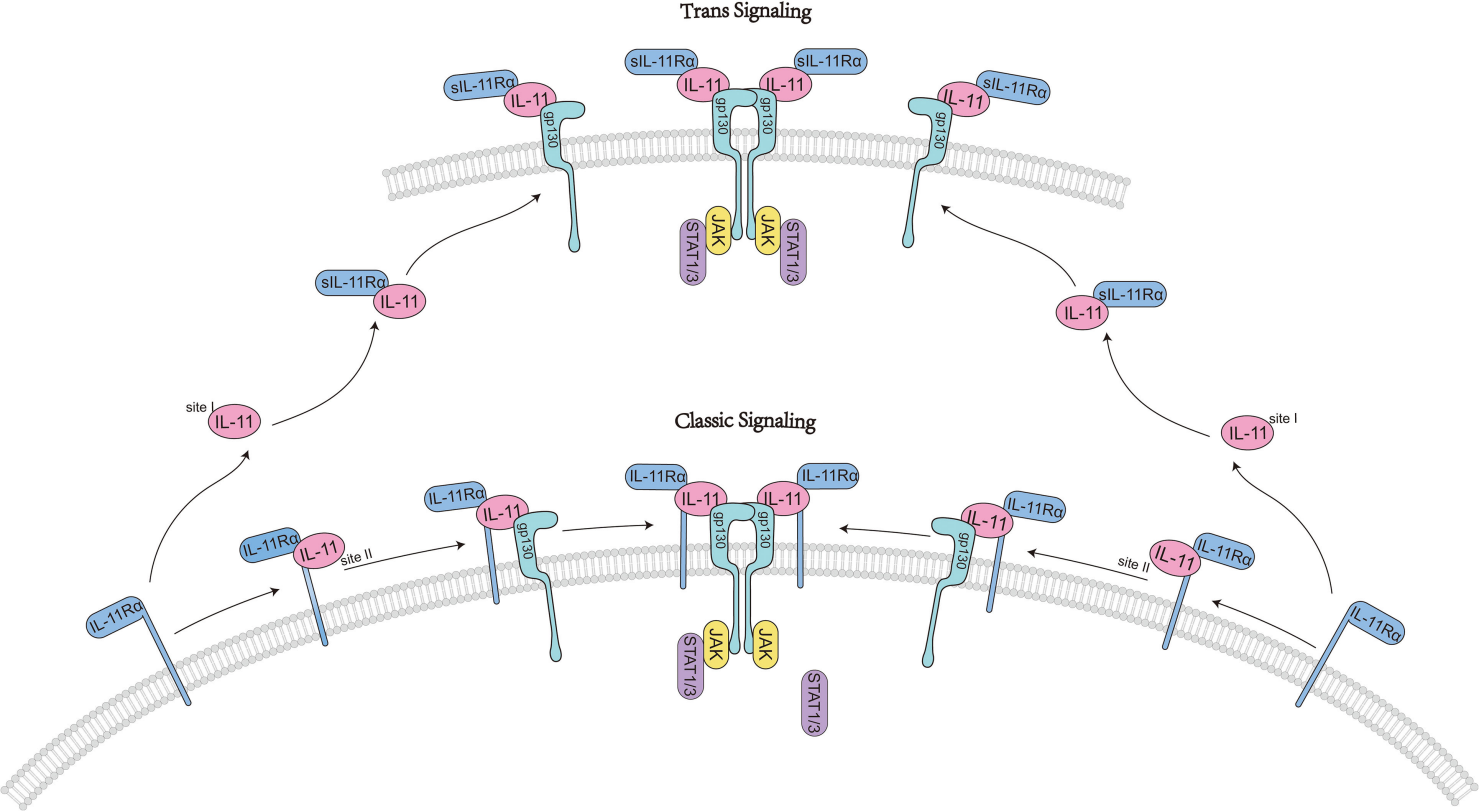
IL11-activated classic signaling pathways.

IL-11 signaling occurs via two pathways: the classical and non-classical pathways. In “classical IL-11 signal transduction,” the formation of the hexameric IL-11 signaling complex on the extracellular membrane leads to the recruitment and activation of JAKs, primarily JAK1, to the cytoplasmic domain of gp130 (13). The activated JAK then phosphorylates tyrosine residues on gp130, affecting the docking, phosphorylation, and activation of signal transducer and activator of transcription (STAT) molecules, specifically STAT1 and STAT3. This mechanism modulates the expression of particular genes associated with distinct biological processes like cell growth, differentiation, and survival (13) (**Figure 2**).

In addition to the JAK/STAT pathway, the IL-11 signaling complex can activate other signaling pathways, including the phosphoinositide 3-kinase (PI3K), mitogen-activated protein kinase (MAPK), and extracellular signal-regulated kinase (ERK) pathways, which also play important roles in cell growth, differentiation, and survival (14, 15). However, their specific mechanisms of action still require further research. Inhibition of AMPK and activation of the mammalian Target of Rapamycin (mTOR) both play very important roles in fibrosis and autophagy (16, 17). IL-11-stimulated ERK/P90RSK activity promotes the phosphorylation of the serine-threonine liver kinase B1 (LKB1), inhibits AMPK and promotes mTOR activation, αSMA expression, and myofibroblast transformation (18, 19). This signaling transduction has been proven to be a common signaling pathway in matrix cells, epithelial cells, and cancer cells (18), providing new ideas and theoretical basis for subsequent fibrosis and cancer-related research and treatment.

Notably, IL-11 signaling can proceed via multiple modalities that expand beyond classical cis-signaling (8). In cis-signaling, IL-11 engages membrane-bound IL-11Rα on the same cell to form a high-affinity complex with gp130 and initiate canonical JAK/STAT and parallel kinase cascades (11). In contrast, trans-signaling involves a complex of IL-11 bound to a soluble form of IL-11Rα (sIL-11Rα), which can engage gp130 on cells that express low or negligible membrane IL-11Rα, thereby expanding the repertoire of responding cells (20, 21). The mechanisms generating sIL-11Rα may include proteolytic cleavage of membrane IL-11Rα by metalloproteases, although the relative contribution of regulated shedding vs alternative splicing remains to be fully defined for IL-11 compared with IL-6R in the IL-6 system (22).

## 3 Role of IL-11 in physiological and pathological processes

IL-11 is expressed in a variety of tissues, including the brain, testes, and hematopoietic organs (23), and plays a significant role in multiple physiological and pathological processes. Under physiological conditions, it is expressed at low levels (24) and is primarily secreted by mesenchymal cells such as epithelial cells, chondrocytes, osteoblasts, leukocytes, fibroblasts, keratinocytes, and synoviocytes (2). IL-11 activates downstream signaling pathways including PI3K/Akt, JAK/STAT1/STAT3, and Ras/Raf/MAPK by binding to IL-11Rα and gp130, thereby promoting the transcription of target genes and participating in various physiological activities (25). It is worth adding that IL-11 cis and trans modes may have distinct biological implications (21). Trans-signaling in the IL-6 system has been associated with pro-inflammatory amplification in contexts such as chronic inflammation (26, 27). By analogy, IL-11 trans-signaling could in principle contribute to pathophysiological responses in cells lacking IL-11Rα, but the physiological dominance and tissue breadth of IL-11 trans-signaling are less well established than for IL-6, with some evidence suggesting that cis-signaling predominates in stromal compartments rich in membrane IL-11Rα (20, 28).

As a potent hematopoietic factor (29), IL-11 can synergize with other cytokines to induce megakaryocyte production (30); it is also involved in the generation of bone marrow, lymphocytes, and erythrocytes (11). In clinical settings such as chemotherapy or other conditions that lead to thrombocytopenia and increased risk of bleeding, IL-11 can reduce the incidence of bleeding events and improve patients’ hemostatic capacity by increasing the plasma levels of von Willebrand factor (vWF) and fibrinogen (31). Furthermore, IL-11 can inhibit adipogenesis (32) and regulate processes such as embryonic implantation, trophoblast invasion, and the formation of a normal placenta (33–35). Additionally, systemic and specific deficiencies in IL-11 lead to a reduction in bone mass and bone formation, accompanied by fat accumulation, glucose intolerance, and insulin resistance (25).

In pathological states, the function of IL-11 is more complex, exhibiting both anti-inflammatory and pro-inflammatory properties. On one hand, IL-11 acts as an anti-inflammatory cytokine. Studies have shown that IL-11 can enhance the secretion of IgG and IgM and promote the differentiation of human B lymphocytes in the presence of helper T cells and monocytes (36). IL-11 may mitigate inflammatory responses in autoimmune diseases by inhibiting the proliferation of T helper type 1 (Th1) cells and promoting the differentiation of Th2 cells (37, 38). IL-11 can suppress the polarization of Th1 cells by affecting T lymphocytes and reducing the production of IL-12 by macrophages (39). Furthermore, IL-11 can exert anti-inflammatory effects by downregulating the activity of macrophages through the inhibition of tumor necrosis factor-alpha (TNF-α), interleukin 1 beta (IL-1β), interleukin 12 (IL-12), interleukin 6 (IL-6), Interferon-γ (IFN-γ), and nitric oxide (NO) production (40–42). Concurrently, Mesenchymal stromal cells (MSCs) can inhibit the ability of IFNγ-secreting CD4 and CD8 cells to produce, thereby preventing T cell-mediated tissue inflammation and tissue damage (43).

More importantly, IL-11 also exhibits pro-inflammatory properties, especially in inflammation associated with cancer. The role of IL-11 in loads of inflammation-related cancers is increasingly gaining attention, as it may promote tumor development and metastasis by affecting immune and stromal cells in the tumor microenvironment (24). In a variety of fibrotic diseases, IL-11 can promote the activation of myofibroblasts, dysfunction of parenchymal cells, and inflammatory responses during the fibrotic process, while inhibiting tissue regeneration (44). Mutations in the IL-11 and IL-11Rα genes are associated with loss of height, osteoarthritis, and craniosynostosis in humans (25). Notably, IL-11 is upregulated in the knee joint tissues of patients with osteoarthritis (45) and in degenerated cartilage (46), and is closely related to disease progression. Notably, inhibition of the IL-11 signaling pathway has been associated with the extension of health and lifespan in mammals. Anti-IL-11 therapy can improve metabolic function in aged mice, reduce the decline in muscle mass and strength, and extend their lifespan (47).

## 4 IL-11 and chronic diseases

**Figure 3:**
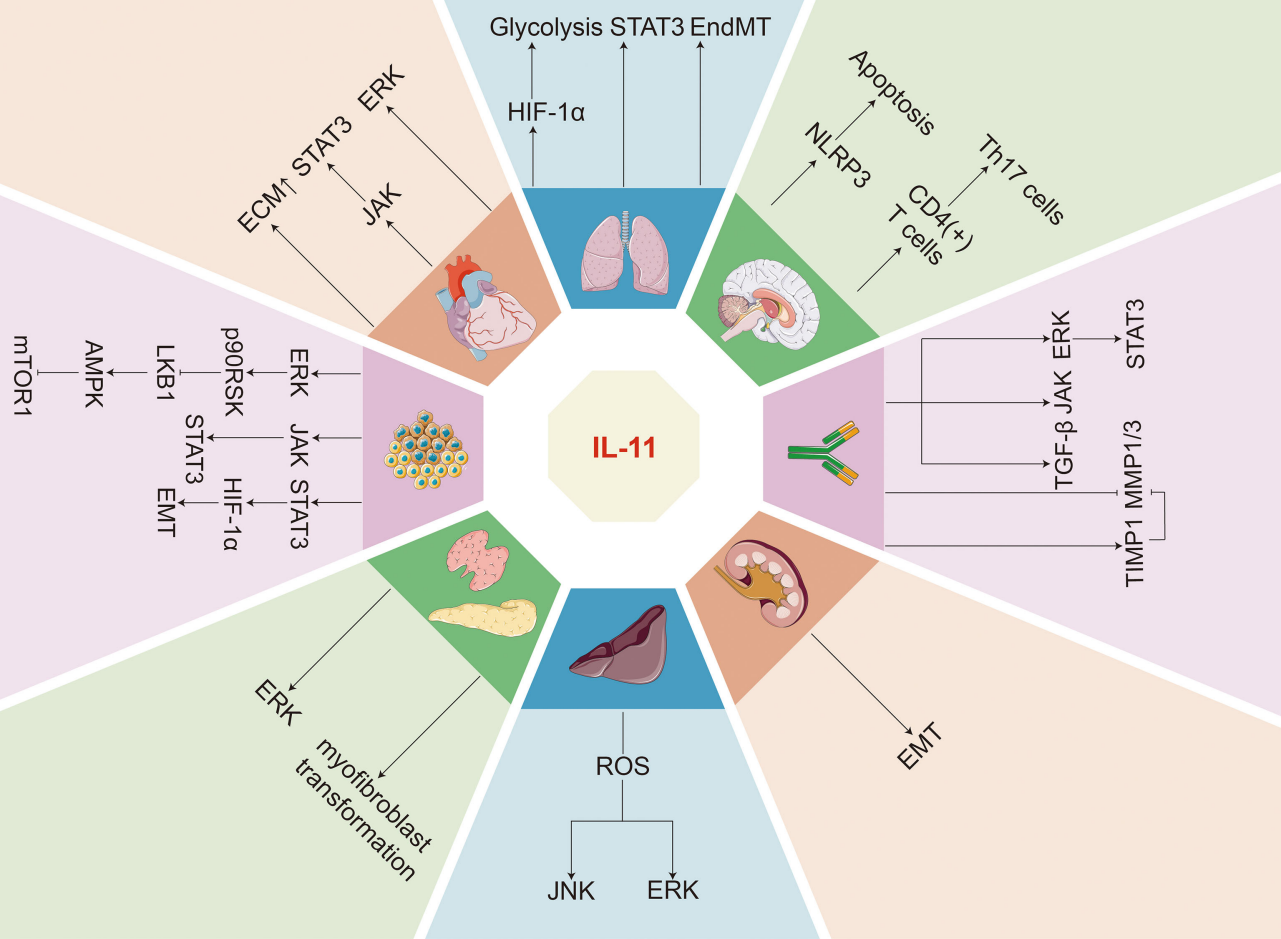
IL11-activated signaling pathways and organ-level pathologies in chronic diseases. IL-11 exerts its effects in chronic diseases by activating signaling pathways such as JAK/STAT, ERK, and PI3K/Akt. Its levels are elevated in various chronic conditions, including cardiovascular, respiratory, and neurodegenerative diseases, where it is involved in inflammatory responses and promotes fibrosis, as well as influencing tissue repair and regeneration. its specific mechanisms of action and impacts vary according to the type of disease.

In chronic diseases such as inflammation, fibrosis, and cancer, the level of IL-11 is significantly higher and is basically in connection with the occurrence and severity of the disease (**Figure 3**).

### 4.1 Cardiovascular diseases

The function of IL-11 in cardiovascular diseases has garnered extensive research attention. IL-11 is a key factor in cardiac fibrosis, with its expression regulated by transforming growth factor beta 1 (TGFβ1) and is involved in the process of cardiac fibrosis (48). It was indicated that IL-11 and IL-11Rα were particularly expressed in fibroblasts and drove a non-canonical ERK-dependent autocrine signaling, which was crucial for the activation of fibroblasts (48). In mouse models, IL-11 did not activate mouse fibroblasts at physiological concentrations (49). However, when IL-11 expression is specifically targeted to the heart and kidneys or when IL-11 is injected, organ fibrosis and functional failure occur, while genetic deletion of IL-11Rα1 can prevent the progression of disease (48). Notably, elevated levels of IL-11 also increase the likelihood of chronic heart failure (CHF) in patients, with higher IL-11 levels correlating with increased expression of N-terminal pro-brain natriuretic peptide (NT-proBNP), indicating a more severe degree of heart failure (50). The reason is that IL-11 activates the transformation of fibroblasts into myofibroblasts, expressing α-smooth muscle actin and secreting extracellular matrix proteins during the process. This leads to excessive deposition of the extracellular matrix, causing myocardial fibrosis and ventricular remodeling, further exacerbating CHF and increasing the risk of poor prognosis (51). Additionally, in coronary artery disease (CAD), hypertension, viral myocarditis, etc., the expression levels of IL-6 are significantly elevated and closely associated with disease progression (52). IL-11, a member of the IL-6 family, has also been found to be significantly higher in CAD compared to non-CAD patients (53), suggesting that similar to IL-6, IL-11 may contribute to the aforementioned chronic diseases, with the specific mechanisms requiring further research and analysis.

IL-11 in the treatment of cardiovascular diseases may also cause adverse reactions in the cardiovascular system, such as water and sodium retention and atrial arrhythmias. Studies have found that as the degree of heart failure worsens, the concentration of plasma IL-11 gradually increases (50). The increase in plasma IL-11 significantly increases the probability of chronic cardiac events (50). Adverse events associated with recombinant human Interleukin-11 (rhIL-11) treatment, such as dyspnea, edema, pleural effusion, and conjunctival congestion, are often considered the result of fluid retention and increased plasma volume (54). Notably, rhIL-11 treatment has been proven to be closely related to atrial arrhythmias, which means rhIL-11 may generate additional risk for patients who have a risk of atrial arrhythmias (44, 54). Injection of IL-11 can directly activate the IL11RA/JAK/STAT3 pathway in cardiomyocytes, leading to acute heart failure (55). Meanwhile, rhIL-11 treatment significantly increases blood pressure and serum BNP in patients, increasing the risk of heart failure (51, 56). Therefore, for patients with water and sodium retention, congestive heart failure, and atrial arrhythmias, especially the elderly, IL-11 treatment should be used cautiously, with regular checks of serum BNP as well as blood pressure and weight changes, and close monitoring for the occurrence of adverse reactions.

Collectively, these results represent that IL-11 acts as a guiding hand in cardiovascular disease and its expression is regulated by TGFβ1 and involved in the cardiac fibrosis process. Both in fibroblasts and in a mouse model of chronic heart disease, IL-11 is specifically and highly expressed with the receptor IL-11Rα, thereby activating signaling and promoting fibrosis and cardiac function failure. In contrast, knockdown of the IL-11Rα1 gene prevented disease progression.

### 4.2 Respiratory diseases

IL-11 plays a key role in the development of multiple chronic lung diseases. By affecting various cellular and signaling pathways, IL-11 makes a big difference in lung function, degree of fibrosis, and inflammatory responses.

IL-11 is associated with the development of pulmonary fibrosis, especially in patients with idiopathic pulmonary fibrosis (IPF), and its increased expression is positively associated with decreased lung function and the degree of fibrosis. As with cardiac fibrosis, IL-11-dependent ERK signaling promotes the differentiation of lung fibroblasts into a mesenchymal-like phenotype and, to some extent, promotes cellular senescence (57). Meanwhile, this pathway further promotes lung fibroblast invasion and metastasis, as well as the expression of Actin Alpha 2, Smooth Muscle (ACTA 2) and collagen (58). Bleomycin can cause oxidative stress, alveolar epithelial cell necrosis, fibroblast proliferation, and infiltration of immune cells, ultimately leading to pneumonia and pulmonary fibrosis (59). IL-11 may increase chronic immune infiltrates and pro-inflammatory gene activation by promoting ERK activation and STAT3 phosphorylation, thereby enhancing fibroblast invasion and aggravating pulmonary fibrosis caused by bleomycin (60). Antibody Neutralization of IL-11 can prevent and alleviate bleomycin-induced lung fibrosis and inflammation (60). In addition, new studies show that IL-11 also causes alveolar epithelial type II cell (AT2) dysfunction and the maintenance of a pro-fibrotic KRT8 alveolar epithelial cells status, which limits the terminal differentiation of AT1 cells and impairs alveolar regeneration (61). Meanwhile, IL-11 can stimulate the transformation of fibroblasts to myofibroblasts, as well as profibrotic KRT8 cells expressing pathological extracellular matrix proteins, leading to pulmonary fibrosis and inflammation (61).

It is noteworthy that IL-11 has a potential value in the prediction of survival and progression of acute exacerbation (AE) in patients with IPF. In IPF patients, serum IL-11 levels are linked with the progression of pulmonary function indicators and AE, suggesting that IL-11 may be a prognostic biomarker and acute exacerbations of IPF (62). It is also noted that, in IPF, IL-11 and IL-11Rα expression are risen in pulmonary arterial hypertension (PAH) and correlated with the severity of pulmonary hypertension (63). IL-11 makes a difference to the development of pulmonary artery remodeling and hypertension by promoting the endothelial to mesenchymal transition (EndMT) process (63). Furthermore, by IL-11 inhibition, fibroblast circulatory and pulmonary accumulation are significantly reduced in animal models of PAH-associated pulmonary fibrosis (64). This finding provides an important rationale for the treatment of PAH-related pulmonary fibrosis in IL-11 (64). Targeting the IL-11 system Targeting IL-11 system (namely targeting IL-11 and IL-11Rα) provides an effective idea and method for the treatment of PAH (65).

Moreover, some studies have explored the pathogenesis of pulmonary fibrosis-related silicosis disease. This study has shown that in crystalline silica (CS)-induced silicosis mice, IL-11 expression increased significantly, activated the ERK pathway and increased hypoxia inducible factor-1α (HIF-1α) expression (66). HIF-1α is a key factor in regulating glycolysis (67). IL-11 promotes mitochondrial glycolytic progression through this ERK-HIF-1α axis, which leads to the development and development of pulmonary fibrosis in silicosis (66).

It is noteworthy that IL-11 also plays an important role in other chronic lung diseases, such as tuberculosis, chronic obstructive pulmonary disease (COPD), bronchiectasis, and asthma. In tuberculosis, IL-11 antibody treatment is effective in reducing neutrophil infiltration and associated inflammatory factor levels and positively feedback downregulates IL-11 mRNA expression (68). In COPD, IL-11 expression is up-regulated in epithelial cells and correlated with the severity of the disease. Meanwhile, genetic susceptibility to COPD is influenced by polymorphisms in the IL-11 gene and IL-11 promoter (69). In addition, patients with gp130 function (homozygous mutation of IL6ST) had significant bronchiectasis and immune abnormalities, indicating that the loss of IL-11 signaling and the development of bronchiectasis were clearly associated (70). In asthmatics, IL-11 expression has been increased significantly in moderate and severe asthma, mainly in airway epithelial cells and myelin basic protein (MBP) -positive eosinophils and was directly associated with disease severity (71, 72). The reason is that IL-11 can promote Th2 polarization and stimulate the production of Th2 cytokines (73). Th2-related responses, such as eosinophilia, mucus hypersecretion, bronchial hyperresponsivenes (BHR), and IgE production, are the basis of asthma pathogenesis (74). It is noteworthy that IL-11 exhibits distinct phenotypes in IL-11Ralpha-null mutant mice and IL-11 overexpressed mice (CC10-IL-11). Compared to wild-type (WT) mice, IL-11Ralpha-null mutant mice demonstrate a significantly reduced Th2-associated response, indicating that IL-11 can markedly enhance airway hyperresponsiveness. In contrast, CC10-IL-11 animals exhibit lower levels of inflammation in tissues and bronchoalveolar lavage fluid, Th2 cell accumulation, and eosinophilia, suggesting that IL-11 significantly suppresses the occurrence of related airway responses (75). The specific mechanism and reasons for this discrepancy still need further study.

### 4.3 Neurological disease

IL-11 and its receptor complex are extensively expressed in various brain structures (76). In the study of multiple sclerosis (MS), IL-11 is thought to promote disease progression by activating the NLRP3 inflammasome, regulating the migration of immune cells to the central nervous system (CNS) (77). IL-11 can also induce the differentiation of naive CD4(+) T cells into Th17 cells and promote the expansion of memory Th17 cells, thereby amplifying the inflammatory response mediated by Th17 in relapsing-remitting MS (78). In the experimental autoimmune encephalomyelitis (EAE) model, anti-IL-11 monoclonal antibody (mAb) inhibits the activation of the NLRP3 inflammasome in peripheral monocytes and the subsequent migration of inflammatory cells, thereby reducing disease severity and neurodegeneration (77). However, IL-11 can also act as a protective factor, promoting the survival and maturation of oligodendrocytes and astrocytes, as well as myelin formation in MS (79). Notably, IL-11 can inhibit L-phosphoserine phosphatase activated by beta-amyloid protein, reducing neurotoxicity, and thus inhibiting the occurrence and development of Alzheimer’s disease (80). Based on this property of IL-11, the increased expression level of IL-11 in early neurodegenerative changes in Alzheimer’s disease (AD) is considered a neuroprotective strategy (81). In addition, studies have confirmed that serum IL-11 levels have a high value in predicting early neurological deterioration in patients with cerebral infarction (82). Research has shown that in a mouse model of cerebral infarction (CI), the expression of IL-11 significantly decreases after CI in mice (83). Similarly, clinical trials have shown that serum IL-11 levels significantly decrease in patients with CI (82). Treatment with exogenous IL-11 can improve neurological function, reduce the volume of CI, and alleviate ischemia-reperfusion injury through anti-inflammatory and antioxidant effects (83).

In summary, the role of IL-11 in multiple sclerosis is very complex. On the one hand, it can aggravate inflammatory responses and disease progression. However, on the other hand, it can protect oligodendrocytes and promote myelin formation. Additionally, IL-11 can reduce neurotoxicity, combat AD and predict neurological deterioration in patients with CI.

### 4.4 Endocrine diseases

IL-11 plays an essential role in lots of endocrine diseases, especially in promoting fibrotic and inflammatory processes. Chronic pancreatitis (CP) is classically identified with exocrine dysfunction and progressive fibrosis (84). Recent research has revealed the key role of the pancreatic clock in the pathological process of CP, indicating that genetic or environmental disruptions to it may exacerbate the fibrotic process and the degeneration of exocrine function (84). Specifically, the absence of Brain and muscle arnt-like 1 (Bmal1) has been shown to lead to uncontrolled fibrotic characteristics of pancreatic stellate cells (PSCs), and through a clock-TGF signaling-IL-11/IL-11Rα axis-dependent manner, reprograms the function of acinar cells (84).

In patients with thyroid-associated ophthalmopathy (TAO), the expression levels of IL-11 in orbital connective tissues and serum are elevated and positively correlated with disease activity (85). IL-11 exacerbates the fibrotic process in TAO by promoting the phenotypic transformation of orbital fibroblasts (OFs) into myofibroblasts (85). Additionally, studies have revealed that IL-11 may function through an autocrine mechanism in TAO (85). In the exploration of common potential target genes for diabetes and kidney stones, researchers have found that IL-11 may be used as a diagnostic biomarker for both conditions (86). For diabetic retinopathy (DR), the IL-11/IL-11Rα signaling pathway may participate in the angiogenic process of proliferative diabetic retinopathy (PDR) (87). Furthermore, it has been discovered that IL-11 autocrine mediated by the MEK/ERK/RUNX2 signaling pathway can activate Müller glial cells, disrupt the blood-retina barrier (BRB), and thus promote the progression of retinal pathology (88).

### 4.5 Kidney diseases

IL-11 is elevated in various kidney diseases in response to diverse injuries, including diabetes, hypertension, ischemia, drugs, infections, obstructive nephropathy, etc (89). Increased levels of IL-11 in urine are associated with nephritis and end-stage kidney disease in patients (89). Overexpression of IL-11 is significant for renal fibrosis; elevated levels of IL-11 can directly induce epithelial-to-mesenchymal transition (EMT), thereby increasing the synthesis of profibrotic mediators (90). The genetic deletion of IL-11Rα can effectively prevent kidney fibrosis in mice, and neutralizing IL-11 antibodies have anti-fibrotic effects (91). Notably, recent research has found that micheliolide can also block the binding of IL-11: IL-11Rα, playing an anti-inflammatory role in diseases related to renal fibrosis (90). Furthermore, IL-11 may be used as a biomarker for the severity of hypertensive nephrosclerosis. Studies have shown that renal IL-11 expression significantly increases in rats with renovascular hypertension and is associated with profibrotic markers and loss of renal function (92). In a mouse model of Alport syndrome (AS), IL-11 levels in the kidney increase as renal failure progresses. Concurrently, IL-11Rα1 is upregulated in stromal fibroblasts and vascular smooth-muscle cells, promoting myofibroblast transformation and driving the occurrence and development of renal fibrosis (93). Notably, IL-11 also plays an essential role in the repair of kidney injury. After kidney damage, the IL-11 signaling pathway may affect the regenerative capacity of the kidney, thereby blocking the promotion of kidney repair and regeneration. In models of chronic kidney disease, anti-IL-11 treatment can promote the proliferation of renal tubular epithelial cells and the regeneration of the parenchyma, reversing fibro-inflammatory conditions and restoring kidney mass and function (94).

### 4.6 Liver diseases

Previous studies generally believed that rhIL-11 possesses anti-fibrotic, anti-inflammatory, and cytoprotective properties in liver diseases (95–99). However, current research findings have primarily focused on the damaging effects of IL-11 on hepatocytes.

Hepatic Stellate Cells (HSCs) are the main cell population responsible for the synthesis of extracellular matrix in the liver, and HSC activation acts as a crucial process in hepatic fibrosis (HF) (100). LX-2 is a human liver cell line that plays a significant role in HF (101). Studies have shown that in patients and mice with hepatic fibrosis (HF), elevated levels of IL-11 further promote the activation and proliferation of LX-2 and induce apoptosis of normal liver cells (LO-2) by activating the ERK and c-Jun N-terminal kinase (JNK) pathways (102).

Another study investigated the role of IL-11 in alcohol-related liver disease (ALD), including alcoholic hepatitis (AH). The study found that serum concentrations and hepatic expression of IL-11 increased with the severity of liver disease, particularly in AH. The study also indicated that serum IL-11 levels above 6.4 pg/mL are an independent risk factor for survival without transplantation in patients with advanced liver disease. Furthermore, anti-IL-11 receptor antibodies (anti-IL11RA) reduced pathological signaling pathways both *in vivo* and *in vitro*, and protected hepatocytes and mouse livers from ethanol-induced inflammation and injury (103). Additionally, IL-11 could regulate hepatocyte metabolism, promoting the transition from non-alcoholic fatty liver disease (NAFLD) to non-alcoholic steatohepatitis (NASH) (104). Concurrently, neutralizing IL-11 antibodies can effectively inhibit liver fibrosis, steatosis, hepatocyte death, and inflammation in NASH mice (49).

In addition, contrary to previous beliefs that IL-11 might improve liver injury induced by acetaminophen (APAP) (95), recent studies have found that IL-11 has a destructive effect on human liver cells (105). In a mouse model of APAP-induced liver injury, excessive APAP administration promotes the upregulation of IL-11 expression, further promoting the production of reactive oxygen species (ROS) and activating the ERK/JNK signaling pathway (105). Anti-IL-11 treatment not only reverses liver injury but also supports liver regeneration, promoting the repair of the liver in damaged mice (105). Stuart A. Cook and others have explained the reasons for the differences between the studies (44). They believe that in mice, rhIL-11 can act as an inhibitor of endogenous mouse IL-11, binding to mouse IL-11rα1, thereby blocking the action of endogenous mouse IL-11. This means that when endogenous IL-11 is elevated in damaged tissues, its action on target cells (such as hepatocytes) is blocked by exogenous and heterologous rhIL-11, and rhIL-11 itself does not activate IL-11 signaling in mice, providing an important theoretical basis for the subsequent use of rhIL-11 in the treatment of related liver injuries (44).

### 4.7 Autoimmune disease

IL-11 plays a significant role in various autoimmune diseases. Compared to patients with osteoarthritis (OA), patients with rheumatoid arthritis (RA) express higher levels of IL-11 in synovial tissue, synovial fluid, and serum (106, 107). The binding of IL-11 to IL-11Rα on endothelial cells can directly induce RA angiogenesis. In addition, the combination of IL-11 and IL-11Rα can promote the migration of RA fibroblasts, thereby enhancing the secretion of pro-angiogenic factors by migrating fibroblasts and indirectly promoting neovascularization in RA (108). Matrix metalloproteinases (MMPs), a family of proteases that have a huge effect on cartilage and bone destruction, have their activity regulated by tissue inhibitors of metalloproteinases (TIMPs) (109–111). Blocking endogenous IL-11 can lead to an increase in TNFα levels, while the exogenous addition of IL-11 can directly inhibit the production of MMP-1 and MMP-3, and upregulateTIMP-1 (107).

It is noteworthy that IL-11 does not play a protective role in all autoimmune diseases. In Systemic sclerosis (SSc) patients, IL-11 is highly upregulated in skin and lung fibroblasts, which is associated with IL-11 dependent ERK pathway activation and TGFβ-stimulated fibroblast proliferation (112). Blocking the JAK2/STAT3 pathway can inhibit the profibrotic effects of IL-11 in SSc (113). Similarly, in patients with Crohn’s disease (CD) or ulcerative colitis (UC), IL-11 is highly upregulated in the colonic mucosa (114). IL-11 shows promise as a novel therapeutic target for IBD patients resistant to anti-TNF treatment (114). Furthermore, Th17 cells have been demonstrated to be directly related to the onset and progression of Experimental autoimmune myocarditis (EAM) (115). IL-6 can induce the occurrence of EAM by affecting the differentiation of Th17 cells (116). Additionally, IL-6 may play a significant role in the progression of autoimmune heart disease by upregulating the C3 complement (117). It can be inferred that IL-11, being in the same family as IL-6, likely has a similar role in experimental autoimmune myocarditis (EAM) as IL-6, but the specific mechanisms still require further research and discussion.

### 4.8 Cancer

IL-11 plays a significant role in the development and progression of cancer. It not only enhances the invasion and metastasis of cancer cells but also impacts the prognosis and recurrence of cancer. Many studies have detailed the increased expression of IL-11 in various types of cancer, including but not limited to breast cancer, lung cancer, gastric cancer, prostate cancer, colorectal cancer and osteosarcoma (118).

Although it has been clear that IL-11 has a significant impact on tumorigenesis and progression, the particular mechanism of action requires additional investigation. First, many studies have confirmed that IL-11-related JAK-STAT3 signaling transduction pathway, played an essential part in cancer development. IL-11 was demonstrated in lung cancer to induce the phosphorylation of STAT3 and to upregulate the expression of the anti-apoptotic proteins Survivin and Bcl-2 in cancer cells (119). IL-11-expressing fibroblasts can further phosphorylate STAT3 through the secretion of IL-11, thereby inducing the activation of colonic fibroblasts and epithelial cells and finally leading to the proliferation of surrounding tumor cells (120). Notably, IL-11/STAT3 signaling can also promote HCC metastasis, and blocking IL-11-STAT3 signaling can inhibit tumor cell proliferation as well as postoperative recurrence of HCC tumors (121).

Additionally, IL-11 can enhance the activity of the ERK/P90RSK pathway, leading to the inactivation of the tumor suppressor gene LKB1, thereby inhibiting the LKB1/AMPK pathway and increasing the expression of mTOR1, which further promotes the occurrence and development of lung cancer (18). The IL-11 and IL-11R system-related PI3K and MAPK pathways have been proven to play significant roles in tumor progression and metastasis (122). Studies have demonstrated that the synergistic interaction between activator protein 1 (AP-1) and Hypoxia-inducible factor 1 (HIF-1) mediates the transcriptional activation of the IL-11 promoter, thereby promoting the survival of hypoxia-associated cancer cells (123). Furthermore, rhIL-11 may also promote the growth of A549 and EMT by activating the STAT3/HIF-1α/EMT axis, which finally drives cancer growth (124).

## 5 Potential applications of IL-11 in therapy

### 5.1 Potential of IL-11 as a therapeutic agent

Under physiological conditions, IL-11 can promote the generation of megakaryocytes, erythrocytes, and platelets. Therefore, rhIL-11 is widely used in the clinic to lessen the need for platelet transfusions with non-myeloid malignancies and treat chemotherapy-induced thrombocytopenia in patients (125, 126). Additionally, the combined use of rhIL-11 with granulocyte colony-stimulating factor (G-CSF) may have synergistic hematopoietic effects, promoting earlier recovery of neutrophils (126). The imbalance of Th1/Th2 cell homeostasis is one of the important pathogenic mechanisms in immune thrombocytopenia (ITP). IL-11 can regulate the balance of Th1/Th2 cells, promote the upregulation of T-bet and the normalization of the ratio of GATA-binding protein 3 (GATA-3), thereby achieving therapeutic effects (37, 38).

Furthermore, IL-11 is a factor that plays a significant part in the process of bone formation and is crucial for maintaining normal bone turnover and preserving trabecular bone mass (127). Currently, IL-11 has shown anti-inflammatory effects in rodent collagen-induced arthritis models (128) and can effectively protect mitochondrial function, along with G-CSF and IL-6, stimulating the growth of radiation-damaged bone marrow (BM) and reducing immune responses to radiation damage (129). Although there are not enough studies about the potential effects of increased IL-11 signaling in the joints of osteoarthritis, it is worth assessing the potential therapeutic efficacy of this treatment in disease models (46).

Given the central involvement of IL-11 in the pathogenesis of chronic diseases, particularly fibrosis and inflammation, there has been growing interest in targeting IL-11 signaling therapeutically (8). Anti-IL-11 strategies are being explored at multiple levels, including neutralizing the IL-11 cytokine itself, blocking its receptor IL-11Rα, and intercepting downstream or trans-signaling events (11). IL-11 inhibitors can specifically inhibit the activation of ERK and SMAD, alleviate pulmonary inflammation, and reverse pulmonary fibrosis, thus playing an important role in anti-fibrotic therapy (48, 58). One of the most advanced IL-11–directed agents is BI 765423, an IL-11 ligand–neutralizing monoclonal antibody currently in Phase 2 clinical evaluation for IPF. This trial (ClinicalTrials.gov Identifier: NCT07036523) aims to assess the safety, tolerability, and preliminary efficacy of inhibiting IL-11 to slow or reverse fibrotic remodeling in the lung. Another strategy involves LASN01, an IL-11Rα–blocking antibody that has entered Phase 1 clinical studies to evaluate its safety and pharmacokinetics (ClinicalTrials.gov Identifier: NCT05331300). By blocking IL-11Rα, this approach aims to prevent both classical and any potential trans-signaling mediated by IL-11, inhibiting downstream profibrotic iland proinflammatory pathways (11). Beyond these clinical candidates, additional IL-11–targeting strategies are being explored in preclinical models (8). Neutralizing antibodies and siRNA approaches against IL-11 have demonstrated efficacy in animal models of cardiac, renal, and hepatic fibrosis, suggesting broad applicability for IL-11 inhibition in various fibrotic diseases (49, 130, 131). These preclinical studies indicate that suppressing IL-11 signaling can reduce fibroblast activation and extracellular matrix deposition, which are key drivers of organ dysfunction in chronic fibrotic conditions (11). Additionally, modulating IL-11 trans-signaling further expands therapeutic opportunities. Although IL-11 trans-signaling is less thoroughly characterized than IL-6 trans-signaling, agents like sgp130Fc (olamkicept), which functionally inhibit gp130-mediated signaling, have shown efficacy in inflammatory bowel disease (IBD) by selectively attenuating pathogenic trans-signaling (132). This clinical success provides a conceptual framework for the future development of selective IL-11 trans-signaling inhibitors (**Table 1**).

**Table 1:** Potential of IL-11 as a therapeutic agent in chronic diseases.

Diseases	IL-11’s role	Therapeutic strategies	Reference
Cardiovascular Diseases	Promotes fibrosis, inflammation, and cardiac remodeling in heart failure	Anti-IL-11 monoclonal antibodies, IL-11 receptor blockade for fibrosis	Schafer S, et al., 2017 (48); Zhuang, T. et al., 1995 (131)
Liver Diseases	Involved in hepatic fibrosis, hepatocyte injury, and NASH progression	Neutralizing IL-11, siRNA targeting IL-11, receptor blockade for liver fibrosis	Widjaja AA, et al., 2019 (49)
Kidney Diseases	Drives fibrosis and EMT in renal tubules	IL-11 inhibition via monoclonal antibodies, siRNA for fibrosis, renal function improvement	Milara J, et al., 2024 (64); Widjaja, A. A. et al., 2022 (93)
Pulmonary Diseases	Contributes to pulmonary fibrosis via fibroblast activation and matrix deposition	IL-11 inhibitors in Phase 2 clinical trials for IPF	Ng B, et al., 2019 (58)
Cancer	Promotes tumor growth and metastasis through stromal activation and inflammation	IL-11 signaling blockade with monoclonal antibodies in cancer models	Cardó-Vila M, et al., 2016 (133); Lewis VO, et al, 2017 (134)
IBD	Exacerbates inflammation and fibrosis in the gut	IL-11 trans-signaling inhibitors to reduce inflammation	Schreiber S, et al., 2021 (132)
Autoimmune Diseases	Modulates immune responses, affects T-cell differentiation and inflammation	Anti-IL-11 therapies to reduce inflammation and promote tissue repair	Curti A, et al., 2001 (39)
Endocrine Disorders	Promotes fibrosis in TAO and chronic pancreatitis	Targeting IL-11 in fibrotic thyroid and pancreas diseases in early studies	Wu P, et al., 2022 (85)

Given the pro-tumor effects of IL-11 in cancer, researchers are developing therapies that inhibit IL-11 signaling, such as targeting the IL-11 ligand and components of its receptor signaling complex (118). Therefore, a team has isolated a synthetic peptide ligand (CGRRAGGSC) that can bind to IL-11R and used it to design a drug candidate, bone metastasis-targeting peptidomimetic (BMTP-11). It has pharmacological activity in preclinical models of lung cancer and patient-derived tumors, inducing cell death in lung cancer cell lines (133). Similarly, BMTP-11 has also been shown to significantly inhibit the growth and metastasis of osteosarcoma, with enhanced effects when used in combination with the chemotherapeutic drug gemcitabine (134). In addition, studies have indicated that IL-11 can constitutively activate JAK2-STAT5 through autocrine mechanisms, leading to platinum drug resistance in ovarian cancer. To address this, the study proposed a new strategy for immune therapy to treat platinum-resistant ovarian cancer mediated by a JAK2 inhibitor (LY2784544). LY2784544 can effectively inhibit JAK2 both *in vitro* and in animals, blocking the platinum-resistant IL-11 autocrine mechanism, thereby sensitizing platinum-resistant ovarian cancer cells to cisplatin (135).

In summary, IL-11 is a is a cytokine with multiple functions that is mainly used in the clinic to treat chemotherapy-induced thrombocytopenia as well as minimize the need for platelet transfusions in patients with non-myeloid malignancies. It can also regulate the Th1/Th2 cell balance and has potential effects on the treatment of ITP. At the same time, IL-11 is crucial for bone turnover and maintaining trabecular bone mass and plays a key part in the process of bone formation. Moreover, although IL-11 has damaging effects in various diseases, its inhibitors, siRNA, and antibody neutralization therapies play an important role in anti-fibrotic therapy. In the future, the therapeutic potential of IL-11 will continue to be explored, especially in the treatment of chronic diseases and cancer.

### 5.2 Challenges in therapeutic strategies and drug design

From a therapeutic perspective, there are both opportunities and challenges in targeting soluble IL-11 vs membrane receptor–dependent pathways (21). Targeting sIL-11/IL-11Rα complexes could theoretically dampen trans-like signaling while preserving beneficial cis-signaling in homeostatic compartments, but this strategy depends on the extent to which trans-signaling actually contributes to disease phenotypes in a given tissue (20, 21). In contrast, antagonizing membrane IL-11Rα directly inhibits both cis and any sIL-11Rα-mediated trans-signaling, which may improve efficacy in contexts such as fibrosis where stromal cells are key effectors (21). Meanwhile, the relative lack of a well-validated trans-signaling assay for IL-11 compared with IL-6 complicates the preclinical to clinical translation of trans-targeted strategies (21).

Furthermore, The potential adverse effects of IL-11 treatment for diseases still require further exploration. Treatment with IL-11 may lead to mild anemia associated with plasma volume expansion. This type of anemia is primarily mediated by increased renal sodium retention without altering creatinine clearance (31). Therefore, closely monitoring the patient’s plasma volume expansion during IL-11 therapy and timely administration of appropriate diuretics can effectively improve anemia caused by IL-11 (31). Moreover, balancing the protective role of IL-11 under physiological conditions and its damaging effects in chronic pathological states is crucial, especially in controlling the dosage and method of IL-11 for therapeutic use. Some studies have utilized mAbs to develop customized ultrasensitive target engagement biomarker detection (136). This method aids in better understanding the design of preclinical studies and the *in vivo* dynamic interactions between IL-11 and anti-IL-11 antibody therapeutic candidates (136). The research also demonstrates how to use customized ultrasensitive target engagement biomarker detection to estimate IL-11 in various disease models, providing support for the determination of effective human dosages and the clinical potential of anti-IL-11 antibody therapy (136). To address the potential cardiotoxicity associated with IL-11 treatment, the development of targeted monitoring methods for IL-11 expression levels may significantly reduce the risk of adverse reactions to IL-11 therapy in the heart.

Furthermore, multimorbidity is a common phenomenon in chronic disease progression (137). Anti-IL-11 therapy has shown safety in early clinical trials and may be applicable for delaying the progression of chronic disease. When considering the use of IL-11 therapy for multimorbidity, its safety and potential therapeutic effects need to be assessed.

## 6 Future directions

Significant advancements have been made in the study of IL-11, yet there are still many deficiencies to address.

IL-11 is produced by a number of cell types, such as stromal cells, parenchymal cells, hematopoietic cells, endothelial cells, or epithelial cells, indicating a diversity in its cellular origins (120). As various chronic diseases progress, there may be differences in IL-11 secretion by different cells. For instance, fibroblasts and endothelial cells both produce IL-11, but their production can also decline with age. Therefore, it is crucial to identify the primary cellular sources driving the pathology of chronic diseases mediated by IL-11, and more work is required to find the main producers of IL-11 in multiple diseases.

Similarly, IL-11Rα is primarily expressed in non-hematopoietic cells, including parenchymal and stromal cells. In stromal cells, IL-11 and IL-11Rα are highly expressed in fibroblasts, suggesting the possibility of autocrine or paracrine actions. Previous studies have shown that the major fibrogenic regulator TGF-β1 can directly induce IL-11 expression. IL-11-IL-11Rα signaling is also required to properly activate fibroblast activation (48). Additionally, as chronic diseases progress, there are typically higher levels of circulating IL-11 cytokines. It is also important to determine whether these higher circulating cytokine levels are involved in the immunometabolic regulation of chronic diseases. Therefore, more work is necessary to further investigate the interaction between IL-11 and its target cells in the context of chronic diseases.

In the progression of chronic diseases, there is often a low-grade inflammatory response, and immune cells are significant in the regulation of this response. Previous research has demonstrated that IL-11 can enhance the production of IL-33, and the associated IL-33 signaling helps regulate the function of regulatory T (Treg) cells and group 2 innate lymphoid cells (ILC2) (138, 139). In fibroblasts, *in vitro* stimulation with IL-11 can activate the ERK and STAT3 signaling pathways, leading to the generation of chemokines that guide immune cell trafficking and cytokines that regulate immune cell activity (140). Recent studies also indicate that anti-IL-11 treatment can significantly reduce CD68+ macrophages in the white adipose tissue of mice (47). Furthermore, in autoimmune diseases, IL-11 can promote the activation of the myeloid NLRP3 inflammasome and further increase immune infiltration in non-lymphoid tissues (77). However, the impact of IL-11 on resident immune cells and specific macrophage subsets in metabolic tissues of chronic diseases remains uncertain. Therefore, more research is needed to determine whether IL-11 has direct or indirect effects on immune cell subsets.

Existing evidence suggests that IL-11 often exhibits a “protective/pro-repair” phenotype in acute injury (141–143), yet during chronic disease courses it becomes more stably embedded within a network that promotes fibrosis, tumorigenesis, and parenchymal cell functional decline. The extent of IL-11’s role in chronic diseases also varies. Importantly, these directionally distinct effects are not necessarily mutually exclusive; rather, they may reflect “functional drift” of the same signaling axis across different temporal windows, cellular niches, and dose- or species-specific contexts (144–146). Mechanistically, one possibility is that fibrosis is a redundant network phenotype driven by dominant upstream cues such as TGF-β, which can sustain profibrotic programs through multiple parallel downstream branches, such that IL-11 can behave as a strong amplifier in some cellular settings but be bypassed in others—consistent with the argument that IL-11–mediated stromal activation may not represent the “master” regulator of TGF-β–driven fibrosis (145). A second, closely related possibility is etiology dependence: in metabolic-liver disease paradigms, lipotoxicity–inflammation–cell death axes and compensatory cytokine/gp130-family signaling may dominate the fibrogenic trajectory, rendering IL-11 signaling comparatively non–rate-limiting even when hepatic IL-11 is induced (144). Third, the apparent contradiction may reflect stage dependence (acute repair versus chronic maladaptive remodeling), where transient injury-induced IL-11/STAT3 signaling could plausibly support short-term adaptation and regeneration, whereas sustained or recurrent injury may shift tissue “goal states” toward matrix accumulation and scar stabilization; notably, IL-11/STAT3 has been reported to limit fibrotic scarring during tissue regeneration, illustrating how directionality can change with biological context and timing (147). Last but not least, cell-type and compartment specificity may matter more than bulk tissue expression: differences in IL-11Rα distribution and baseline gp130/STAT activity across parenchymal versus stromal compartments could mean that IL-11 sometimes marks an inflammatory–stromal state without being an obligate collagen driver, as suggested in systemic sclerosis where IL-11 expression is caspase-1–dependent yet did not increase collagen deposition in the reported setting (146).

Currently, several clinical trials targeting IL-11 or its receptor to treat fibrosis are underway (16). However, IL-11 possesses pleiotropic homeostatic functions. For instance, loss-of-function mutations in IL-11 or IL-11Rα in humans have been linked to decreased height and the formation of craniofacial abnormalities (148); astrocyte-derived IL-11 has also been proven to promote the survival of oligodendrocytes and myelin formation (79). Long-term use of IL-11 antibody neutralization therapy may have unintended consequences, and further research is required to understand the polymorphism breadth of IL-11 in the general population from multiple dimensions, including the structural activation of IL-11 and its receptor, transcriptional regulation, epigenetic regulation, protein regulation, and post-transcriptional regulation of IL-11. It is crucial to understand how these factors modulate chronic diseases, health-related parameters, or inflammation.

## 7 Conclusion

IL-11, as a pleiotropic cytokine, demonstrates its significance in the context of chronic diseases. Our comprehensive analysis emphasizes the complex interplay among IL-11, various physiological processes and chronic diseases, highlighting IL-11’s potential as a biomarker and therapeutic target. The development of targeted therapies that can modulate IL-11 signaling offers new treatment strategies for chronic diseases. With our understanding of IL-11 in chronic diseases and deepening, its potential in preventing, treating, and managing a broad range of health conditions becomes increasingly promising.
